# Event related desynchronization-modulated functional electrical stimulation system for stroke rehabilitation: A feasibility study

**DOI:** 10.1186/1743-0003-9-56

**Published:** 2012-08-16

**Authors:** Mitsuru Takahashi, Kotaro Takeda, Yohei Otaka, Rieko Osu, Takashi Hanakawa, Manabu Gouko, Koji Ito

**Affiliations:** 1 Terumo Corporation, 1500, Nakai-machi, Ashigarakami-gun, Kanagawa, 259-0151, Japan; 2 ATR Computational Neuroscience Laboratories, 2-2-2 Hikaridai, Seika-cho, Soraku-gun, Kyoto, 619-0288, Japan; 3 Clinical Research Center, National Hospital Organization Murayama Medical Center, 2-37-1 Gakuen, Musashimurayama-shi, Tokyo, 208-0011, Japan; 4 Tokyo Bay Rehabilitation Hospital, 4-1-1 Yatsu, Narashino-shi, Chiba, 275-0026, Japan; 5 Integrative Brain Imaging Center, National Center of Neurology and Psychiatry, 4-1-1 Ogawahigashi-cho, Kodaira-shi, Tokyo, 187-8502, Japan; 6 Department of Mechanical Engineering and Intelligent Systems, Faculty of Engineering, Tohoku Gakuin University, 1-13-1 Chuo, Tagajo-shi, Miyagi, 985-8537, Japan; 7 College of Science and Engineering, Ritsumeikan University, 1-1-1 Nojihigashi, Kusatsu-shi, Shiga, 525-8577, Japan

**Keywords:** Brain Computer Interface (BCI), Brain Machine Interface (BMI), Hemiparesis, Hemiplegia, Motor functional recovery

## Abstract

**Background:**

We developed an electroencephalogram-based brain computer interface system to modulate functional electrical stimulation (FES) to the affected tibialis anterior muscle in a stroke patient. The intensity of FES current increased in a stepwise manner when the event-related desynchronization (ERD) reflecting motor intent was continuously detected from the primary cortical motor area.

**Methods:**

We tested the feasibility of the ERD-modulated FES system in comparison with FES without ERD modulation. The stroke patient who presented with severe hemiparesis attempted to perform dorsiflexion of the paralyzed ankle during which FES was applied either with or without ERD modulation.

**Results:**

After 20 minutes of training, the range of movement at the ankle joint and the electromyography amplitude of the affected tibialis anterior muscle were significantly increased following the ERD-modulated FES compared with the FES alone.

**Conclusions:**

The proposed rehabilitation technique using ERD-modulated FES for stroke patients was feasible. The system holds potentials to improve the limb function and to benefit stroke patients.

## Background

Stroke is a leading cause of neurological disability among adults and often leads to functional deficits in motor control. Functional electrical stimulation (FES) of muscle is known to be one of the effective methods of improving motor function [1]. For the FES technique, synchronization of motor intention and the stimulation timing is important to benefit rehabilitation [2]. Cauraugh et al. used an electromyogram (EMG)-triggered FES to synchronize the motor intent and FES, and reported benefits of this paradigm compared with non-triggered control groups [3]. In the EMG-triggered FES, electrical stimulation is voluntarily triggered by the residual muscle activity in the affected muscle [4]. Significant improvement in motor function of a paralyzed limb has been observed in stroke patients using this system [5-9]. Recent studies have developed new FES systems which control not only the timing but also the intensity of electrical stimulation in a direct proportion to the voluntary EMG [2,10]. The FES systems are triggered or controlled by motor intent detected by voluntary EMG of an affected limb, and, therefore, they are not applicable in severely disordered patients in whom surface EMG is not consistently detectable or it does not coexist with motor intent. For such patients, there are limited therapeutic options currently available.

As a tool to detect motor intent, brain computer interface (BCI) systems based on event-related desynchronization (ERD) of electroencephalogram (EEG), which is interpreted as desynchronized activities of the activated neurons, have been viewed with an interest by neurorehabilitation researchers. These systems can be applicable to severely affected patients whose EMG is undetectable because motor intent is detected from brain activity. Parasad et al. gave visual feedback when the ERD was successfully detected to evaluate the engagement of motor imagery in upper limb recovery [11]. Several other groups have combined BCI with robotic or FES systems to provide a proprioceptive feedback [12-17]. Some of these studies have led to improvements in the upper limb function of stroke patients, although the studies are typically lacking the appropriately matched controls. These systems provide a binary feedback as a measure of success or failure in the motor imagery training after the imagery period is completed. Considering the activity-dependent and signal timing-dependent plasticity of the brain, improving the synchronicity and correlation between motor intent and stimulation may increase the chance of inducing changes in the brain, especially following neurological deficit such as stroke [18-20].

In this study, we developed an ERD-modulated FES system that has better temporal synchronicity and intensity correlation with the motor intent than the previously reported binary feedback systems. As a feasibility study of the ERD-modulated FES system, we applied this system to the affected lower limb of a hemiparetic stroke patient and observed short-term functional improvement after the training using ERD-modulated FES compared with the FES alone.

## Methods

### Participant

A hemiparetic patient (male; 55 years old) with a first-ever ischemic stroke was recruited at the three chronic time points (29, 30 and 34 months after stroke onset). The patient had an infarct in the right brainstem and presented with severe left hemiparesis. His voluntary lower limb movement was not consistently detectable. The score of SIAS foot-pat test [21,22] was either 0 (no voluntary movement is noted) or 1 (minimal movement is noted, but the foot is not lifted from the floor in the sitting position), and varied from day to day. The patient was able to walk independently using an ankle-foot orthosis and a cane. Mini-Mental State Examination score was 29 out of 30. This study was approved by the Ethical Committee of Tokyo Bay Rehabilitation Hospital and was performed after obtaining written informed consent from the patient.

### ERD-modulated FES system

**Figure 1:**
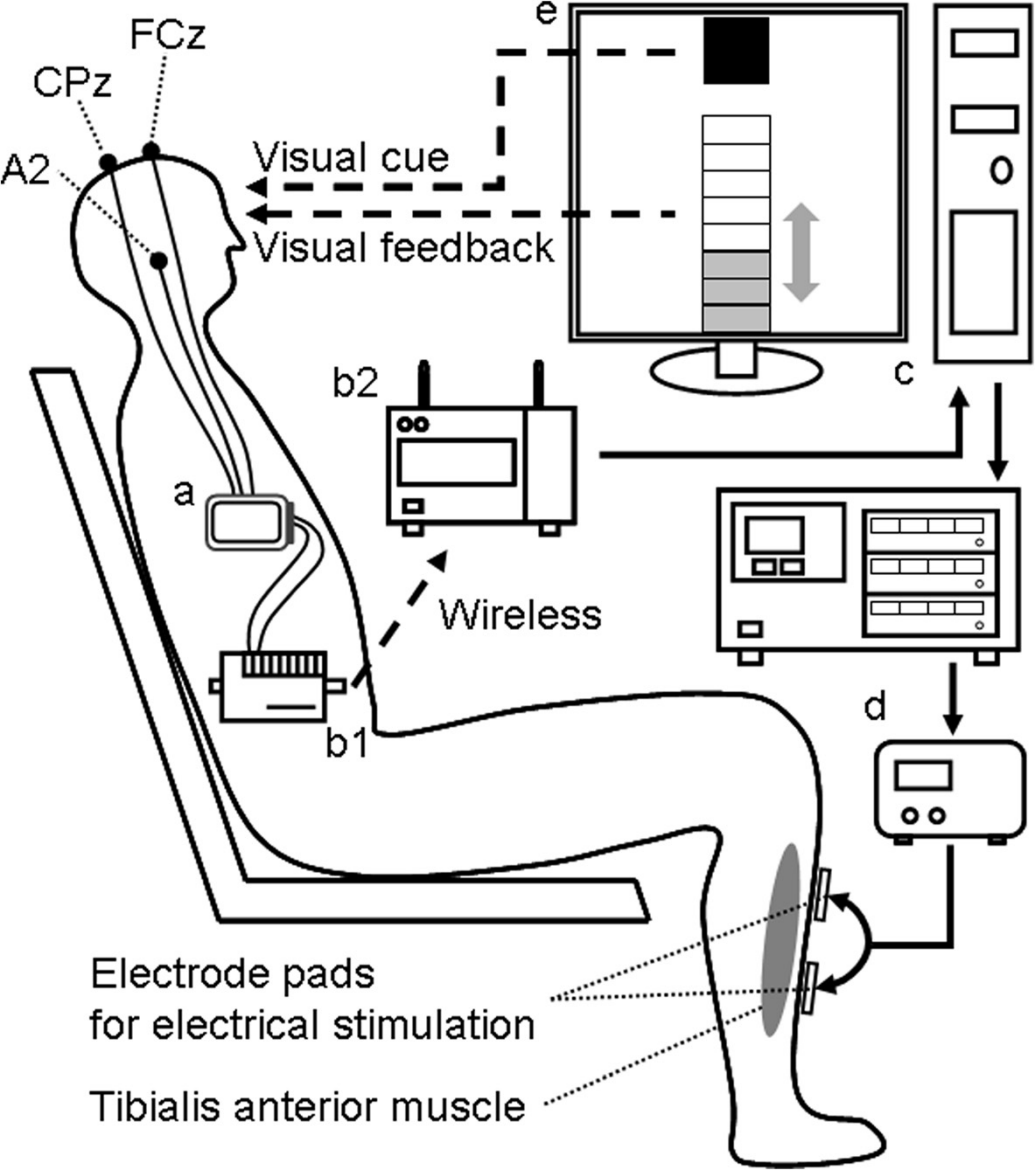
**Schematic of the experimental system.a**: Active electrode system, 3 filled circles: positions of EEG electrodes. **b**: Wireless multi-telemeter system, **b1**: sender, **b2**: receiver. **c**: Computer with A/D-D/A converter. **d**: Electrical stimulator and isolator for FES. **e**: The filled square presented as a cue during the task period. The 8-steps bar height denoted the FES amplitude intensity.

The patient was in the seated position and was instructed to repeatedly attempt dorsiflexions of his paretic ankle joint at a comfortable pace during the cue presentation, which was presented as a red filled square at the top of 17-inch LCD display located in front of the patient approximately 1 m apart (Figure 1e).

For the ERD recording, two active scalp electrodes referenced to the right ear (A2) were put onto FCz and CPz according to the international 10–10 system [23]. The EEG was detected using an active electrode system (g.GAMMAsys, g.tec medical engineering Gmbh, Austria), delivered using a wireless multi-telemeter system (WEB-5000, Nihon Kohden, Tokyo, Japan), and was digitized at 256 Hz sampling frequency (Figure 1a,b,c). The electric potential difference between the two scalp electrodes (bipolar method) was analyzed every 500 ms with the fast Fourier transformation to detect β band (24–26 Hz) power during the cue presentation, and ERD appearance was defined when the β band power was below the predetermined threshold (see ***Preparation experiment***).

**Figure 2:**
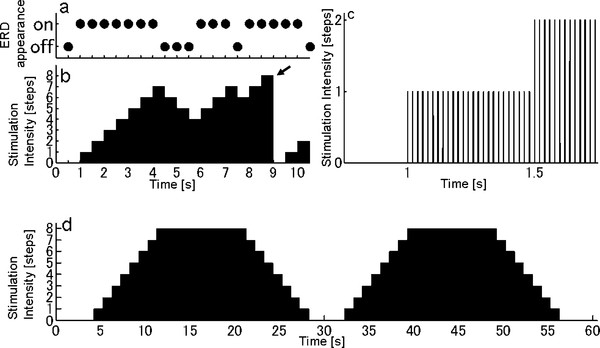
**Temporal sequence of ERD.a**: An example of ERD appearance. The ERD appearance was determined every 500 ms in the intervention experiment. **b**: An example of stimulation sequence in the intervention experiment, which is modulated by ERD appearance shown in (a). The amplitude of stimulation intensity changed dependent on the ERD appearance. When the amplitude reached the maximum step, the current was returned to zero (arrow). **c**: An enlarged view of (b). The amplitude of current pulse train (50 Hz rectangle pulses; 1.2 ms width) was modulated. **d**: For FES training without ERD (control experiment), the stimulation sequence consisting of two trapezoidal forms was used.

For FES of the tibialis anterior muscle to induce dorsiflexion of ankle joint, 74 mm x 47 mm silicone rubber pad electrodes were placed 10 cm and 20 cm distal to the tip of the left fibula. Electrical current pulses (50 Hz rectangle pulses; 1.2 ms width, Figure 2c) were generated by a digital stimulator (SEN-8203, Nihon Kohden, Tokyo, Japan) (Figure 1d) and applied to the pad electrodes via an isolator (SS-104 J, Nihon Kohden, Tokyo, Japan). The ERD appearance was determined every 500 ms, and the magnitude of the FES current was increased step-by-step (each step = maximum current / 8) when the patient successfully showed ERD (Figure 2a,b). Oppositely, the magnitude was decreased step-by-step when the ERD disappeared. The amplitude of FES current that gave the maximum muscle contraction was determined as the maximum current (approximately 8.0 mA). The minimum FES current was set at 0 mA. When the patient attained the maximum FES current (maximum bar height), the current was returned to zero (Figure 2b arrow) for the safety of current stimulation. The step-by-step change of FES current intensity was also visually presented as a step-by-step change in the height of a bar on the LCD display to provide additional feedback to the patient (Figure 1e) to make the patient can know the slight appearance of ERD even when the power of FES current was below a threshold of patient’s sensation.

### Preparation experiment

To predetermine the threshold of the ERD appearance, the patient participated in a preparation experiment 29 months after the stroke onset, and the EEG recording was performed without the FES. The patient was instructed to repeatedly attempt dorsiflexions of his paretic ankle joint at a comfortable pace during the cue presentation, which lasted for 1 minute (defined as the ‘task period’) followed by 20–30 s resting period. A total of 20 blocks were performed. The β band power during the task period and resting period were calculated for each block and they were averaged over all the blocks. The mean value between the averaged β bands during the task and resting periods was set as the threshold of the ERD appearance for the following intervention and control experiments.

### Intervention and control experiments

To examine the effect of the ERD-modulated FES, the patient performed a training of ankle extension under the following two conditions. Intervention experiment (executed 30 months after the stroke onset): FES amplitude was modulated with ERD. Control experiment (executed 34 months after the stroke): FES was applied without ERD modulation. In each experiment, the patient performed 20 blocks of repeated attempts at ankle extension (same as ***Preparation experiment***). In the control experiment, the predetermined two trapezoidal wave pattern (Figure 2d), which is unrelated to motor intent, was used as the FES stimulation. The templates consisted of 2 trapezoidal waves (totally 1 minute): base level (0 mA, 4 s), rising (7 s), high level (10 s), and falling (7 s). Since the stimulation pattern in the intervention experiment was changed step-by-step (see ***ERD-modulated FES system***), the step-wise trapezoidal wave pattern was used for the control experiment. The maximum current in the control experiment was set at 8.1 mA which is an approximately equal value as that in the intervention experiment.

### Evaluations of motor functions

Before and after the training in each experiment, the patient executed assessment sessions, during which, the patient was required to repeatedly attempt dorsiflexions of his paretic ankle joint with near maximum contraction at a comfortable pace for 5 s followed by 5 s resting period for 30 trials.

Using two-bar (10 mm spacing) type surface electrode (DE-2.1, DELSYS Inc. Boston, U.S.A.) placed onto 15 cm distal to the tip of the fibula, EMG signal was recorded from the paretic tibialis anterior muscle (Bagnoli, DELSYS Inc. Boston, USA). It was delivered through the wireless multi-telemeter system, and was digitized at 256 Hz sampling frequency. The EMG data were rectified and smoothed out with a 0.5 s (128 points) moving average. We focused on the peak EMG amplitude during 0.5-2.5 s after the onset of dorsiflexion. Because training under the two experiments were executed on different days, we normalized the peak EMG amplitude of assessment after training in each experiment by dividing the amplitude by a mean peak EMG amplitude obtained from 30 trials before training in that experiment.

The mean of the normalized peak EMG over 30 trials was compared between the two experiments (with and without ERD modulation) by Welch's t-test because the two conditions had unequal variances.

In the assessment session after the training in each experiment, the position of the markers attached to the center of the rotation of knee, ankle, and toe joints of the paretic lower limb were also recorded, using Optotrak 3D motion capture system (Certus, Northern Digital, Waterloo, Canada) at 100 Hz sampling frequency. We calculated the maximum range of motion (ROM) of ankle joint during dorsiflexion for each of 30 trials after the training, and compared the mean of the maximum ROM between two experiments by Welch's t-test because of unequal variances.

The signal processing and statistical analyses were performed using Matlab R2007b (MathWorks, Natick, MA, U.S.A.).

## Results

**Figure 3:**
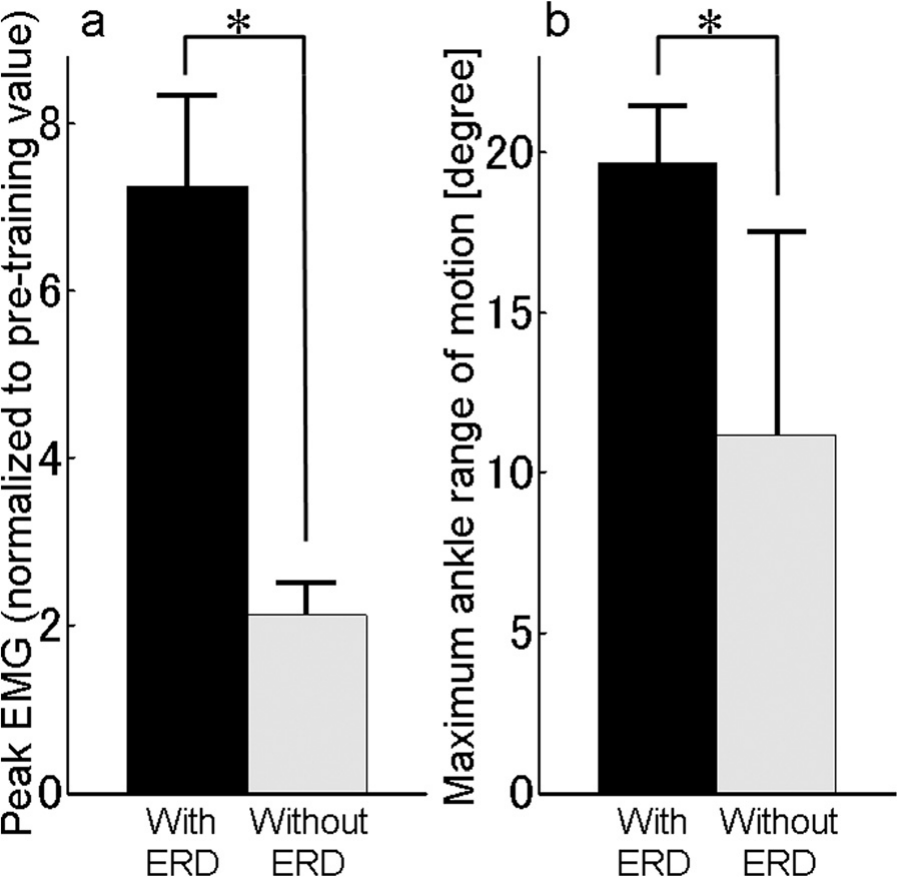
**EMG and maximum angle range of motion.a**: Mean of peak EMG in post-training which was normalized to pre-training. **b**: Mean of maximum range of motion of the paretic ankle after the training. Error bars: SD. Asterisk: *p* < .001. Black bar: intervention experiment (ERD modulated FES). Gray bar: control experiment.

Figure 3a shows the mean ± standard deviation (SD) of the normalized peak EMG under the two experiments (FES with and without ERD). In both experiments, the EMG amplitudes increased after the training (7.2 ± 1.2 for FES with ERD; 2.1 ± 0.2 for FES without ERD). The normalized peak EMG observed under assessment session in intervention experiment was significantly larger than that in control experiment (*p* < .001).

The means (±SD) of the maximum ROM of the paretic ankle in the assessment session after training are shown in Figure 3b (19.7 ± 1.7 degrees for FES with ERD; 11.1 ± 6.4 for FES without ERD). The maximum ROM of the paretic ankle observed after the FES training with ERD was significantly larger than that after FES without ERD (*p* < .001).

## Discussion

This study proposed a new BCI system of FES for stroke rehabilitation, which has better temporal synchronicity and intensity correlation with the motor intent than the previous BCI studies [11-17]. We applied this system to the affected lower limb of a hemiparetic stroke patient to test the feasibility.

When the patient repeatedly attempted dorsiflexions of his paretic ankle joint during the cue presentation, the system successfully detected the ERD appearance, and modulated the amplitude of FES current. Neither short-term (within day) nor long-term (across 4 months) harmful effects of the proposed system were observed in the patient. We observed short-term improvements in EMG of the paretic tibialis anterior muscle and in ROM of the affected ankle just after the training using the ERD-modulated FES compared with that using FES alone. It is crucial to note that this study is only from single stroke patient who received training only once. Concentrated and/or continuous training may be necessary to provide long-term improvements because the case series studies of EMG-triggered FES [4], of contralateral homonymous muscle activity-modulated FES [24], or of ERD-triggered mechanical orthosis which extend the paretic fingers [14,16] showed significant functional improvements after several weeks training. More studies that examine patients with different lesion locations as well as patients with different stages of recovery, utilizing a larger sample size, are necessary to evaluate the capabilities of the proposed system.

Previous EMG-triggered or EMG-modulated FES trainings [3] were unable to target the patients with severely paralyzed muscles. Our system is applicable to those patients who do not have residual motor function in the affected limb. There are several advantages to the current system over the previously proposed BCI rehabilitation systems. First, by combining FES, our system provides proprioceptive and somatosensory feedback in addition to visual feedback, which will likely augment the effect. In addition to the muscle contraction elicited by orthodromic activation conduction, FES can induce antidromic impulses in the motor nerve fibers, which may support the rewiring of the neuron by coincident voluntary motor commands through a Hebb-type modifiable synapse [25]. Assuming that temporal and functional coupling is essential to induce plasticity, the improved temporal synchronicity and correlation between ERD and FES put forth by the current system is expected to be a superior platform for promoting recovery, compared with other systems that give delayed binary feedback after each trial.

The proposed system has several hurdles for clinical use because the system uses EEG to detect the motor intent. EEG signals are too noisy to allow recording of cerebral activity during movement. Therefore, the participant's body movement has to be restricted. The detectability of motor intent using ERD is lower than that using EMG. In addition, ERD of severely affected patient and/or at chronic phase may differ from those of a healthy person.

With respect to BCI training, there is still controversy as to whether we should ask patients to attempt to move or to imagine movement to promote recovery. Because motor imagery necessitates suppression of actual movement, in the current study, we asked the patient to attempt to move so that the actual motor command would reach as far as possible into the neural networks in the brain. On this point, further investigation is needed to establish the utility of BCI rehabilitation in stroke patients.

## Conclusion

We confirmed the feasibility of short-term training using a novel rehabilitation technique that combines ERD and FES. This study extended the therapeutic options of FES rehabilitation to the severely disordered patients who were not applicable for volitional EMG-controlled FES. The results suggest a positive outcome in comparison to FES without ERD. The coupling between motor intent and stimulation may have an effect by inducing neural plasticity. Investigations utilizing a larger sample size and range of clinical cases are required in to further validate the use of this technique in stroke rehabilitation.

## Abbreviations

BCI, Brain computer interface; EEG, Electroencephalogram; EMG, Electromyogram; ERD, Event-related desynchronization; FES, Functional electrical stimulation; ROM, Range of motion; SIAS, Stroke impairment assessment set.

## Competing interests

The authors declare that they have no competing interests.

## Authors’ contributions

MT and KT led this work, designed the experiment, performed the measurement of the patient, signal processing and statistical analysis, and wrote the manuscript. YO recruited the patient from the hospital and performed the measurement of the patient. YO, RO, TH, MG, and KI gave expert guidance for the experimental evaluation and manuscript writing. All authors read and approved the final manuscript.
